# Pathways to the therapist paragon: a decolonial grounded theory

**DOI:** 10.3389/fpsyg.2023.1185762

**Published:** 2023-06-29

**Authors:** Jude Bergkamp, Maeve O’Leary Sloan, Jack Krizizke, Malea Lash, Noah Trantel, Jason Vaught, Tessa Fulmer, Ilana Waite, Abigail M. Martin, Cynthia Scheiderer, Lindsay Olson

**Affiliations:** Antioch University Seattle, Seattle, WA, United States

**Keywords:** privilege, psychotherapy, microaggressions, ethics, imposter syndrome, education

## Abstract

**Introduction:**

While many professional associations within clinical and counseling psychology have made an aspirational call for clinician awareness of social position, there is a lack of research into how socially-conferred privilege impacts psychotherapy. Specifically of interest is the differences in race and gender within the therapeutic dyad, in which there is a BIPOC (Black, Indigenous, and Persons of Color)/white^1^ or male/female-identified dynamic.

**Method:**

The authors utilized a Grounded Theory approach to analyze qualitative interviews with practicing psychologists to construct a process model regarding how socially-conferred privileged identity domains impact the therapeutic relationship and the participants’ professionalization process.

**Results:**

The analysis identified the core conceptual theme of the Therapist Paragon, representing an idealized version of what a perfect therapist should be. This replicated the foundational figures of our field - primarily older, white men. The process model consisted of two distinct pathways toward the Therapist Paragon, one for BIPOC psychologists and one for white psychologists. The female BIPOC pathway consisted of imposter syndrome, persistent feelings of inadequacy, and tendencies to over-credential their professional identity to seek credibility in the eyes of clients and colleagues. The white pathway consisted of down-playing whiteness and attempting to initially modify behavior toward client cultural norms.

**Discussion:**

The results point to a distinct professionalization and practice process for BIPOC psychologists compared to white counterparts. This dynamic may have implications beyond increasing support for BIPOC clinicians specifically, but instead indicate a lack of acknowledgement of the psychological impact of socially-conferred privilege in the psychotherapy enterprise overall. Recommendations are offered for revisions to training models, continuing education, and supervision/consultation.

## Introduction

Social privilege has been increasing in the general discourse over the past decade, making its way into politics and social media (Ross, 2016; Haines, 2019; Asare, 2021). Yet, academic research on the topic has been sparse and mostly resides in the fields of sociology and women’s studies (McIntosh, 2017). Research on individual’s experiences and understanding of social privilege allows scholars and practitioners alike an interesting avenue through which to address systems of oppression and inequality. However, literature reviews today turn up little information regarding individuals’ experiences and understandings of social privilege. While a number of researchers in the fields of sociology, education, and psychology have attempted to address this lack of knowledge of systems of power and privilege (Tatum, 1994; Ferber and Herrera, 2012; Case and Cole, 2013; DiAngelo, 2016; Atkins et al., 2017; Fors, 2018; Johnson, 2018), few of these studies have specifically looked into how social privilege may affect therapeutic practice. Furthermore, there are no studies that address how social privilege might specifically affect doctoral level practice.

American Psychological Association (APA) (2017a,b), declared their aspiration to view psychology through a social justice lens. With this declaration, the APA committed to the process of critically examining the impact historical and modern systems of privilege and oppression have had on the field. While these aspirations are well-intentioned in nature, there remains a lack of guidance regarding the enactment of this paradigm shift within psychotherapy (Abe, 2019).

In this article, we begin with a historical review and definition of social privilege, address issues of intersectionality, and social location, then summarize the current literature related to privilege in psychotherapy. Then we will provide a description of the research design and general principles of grounded theory. The results section will provide common and distinct themes from the date, and the emergent grounded theory. Finally, implications for graduate education, continuing education, and ethics are offered.

## Literature review

### Social privilege

The concept of social privilege can be traced back to the beginning of the 20th century. In 1903, W.E.B. Du Bois published a book entitled, *The Souls of Black Folk*, in which he described the differential experience, or the “double consciousness,” that white and Black people experience as they move through the world. Specifically, Du Bois (1935) noted that although both impoverished white and Black people received low wages for their work, the white workers also received social benefits that were denied to their Black counterparts. Du Bois (1935) termed these benefits “wages of whiteness,” and explained to readers that these wages could only exist as long as Black individuals were oppressed.

Du Bois’ initial definition has evolved over time to address the highly complex praxis of social privilege. Helms (1984) echoed DuBois’ work and developed a five stage White Racial Identity Model, in which she explicitly recognized that because all individuals live in a racialized world, white people’s identities are therefore inherently influenced by racism. This sentiment was furthered by McIntosh (2013), who outlined many invisible ways in which white individuals benefit from oppressive systems. She referred to this concept as white privilege, which can be further understood as an invisible, unearned “knapsack” of benefits and assets: an unquestioned, unearned set of privileges afforded to white people. These privileges contribute to a sense of belonging, physical and emotional safety and wellbeing, general protection from harm, access to opportunity, and ability to remain blind to the impact of race, ethnicity and culture without penalty. McIntosh acknowledged the highly complex and hierarchical nature of white privilege, which can be enhanced depending on one’s sex, sexual orientation, physical ability, age, nationality, or religion. Case and Cole (2013) termed these interlocking hierarchies as “automatic, unearned benefits bestowed upon perceived members of dominant groups based on social identity” (p. 2). Not only is privilege associated with systems of dominance and power, but it is also associated with one’s social identity.

Black and Stone (2005) provided an all-encompassing five domain definition of social privilege. First, they asserted that privilege is a special advantage, which is neither common nor experienced universally. Second, privilege is not earned by effort but socially granted to someone irrespective of individual labor or talent. Third, privilege is a right or an entitlement that is often associated with one’s status and rank. Fourth, individuals utilize their privilege for their own benefit and often at the expense or detriment of others.

Black and Stone’s (2005) fifth and final assertion was that social privilege often exists outside of someone’s conscious awareness. This invisibility, which is evoked within McIntosh’s knapsack metaphor, can also be understood as a form of dysconsciousness. The term dysconsciousness, coined by King (1991), has been adopted to describe lack of awareness of one’s privilege. Dysconsciousness allows those with privilege to receive the benefits of their social location without questioning the systems of power that keep them privileged through the exploitation of marginalized groups. The very nature of social privilege being invisible presents an interesting quandary to those who want to study the concept: how can we study something that we cannot see? While the concept of critical consciousness was first pioneered by Freire (1970) and Heaney (1984) expanded it to describe the active effort one must take to acknowledge their own social privilege, actively judge and critique social order, and refrain from uncritical acceptance of the status quo.

The present study adopts Black and Stone’s understanding of social privilege, while also acknowledging the developmental nature of how human beings come to understand it. Bergkamp et al.’s (2022a,b) Model of Integrating Awareness of a Privileged Social Identity (MIAPSI) offers individuals a way of understanding the idea of social privilege awareness as an active, ongoing, and ever evolving process. According to MIAPSI, social privilege awareness is developmental in nature and occurs in a non-linear and cyclical fashion. Specifically, this includes four phases that individuals move through as they develop their own social privilege awareness: Critical Exposure, Identity Threat, Identity Protection, and Reconciliation. The authors also noted that throughout these four phases, three conducive factors may encourage and help to evolve the development of one’s own privilege awareness. These conducive factors include cognitive scaffolding, interpersonal safety, and intrapersonal safety.

### Intersectionality

Crenshaw’s (1989) theory of intersectionality challenged dominant, single-axis theories which viewed power in terms of “have” and “have nots.” These single-axis frameworks failed to recognize the many “multiply burdened” (1989, p. 14) individuals in this world who possess multiple marginalized social identities, for example Black women. To regard Black women’s social identities in terms of one category or another is insufficient. They should instead be acknowledged as having their own intricate and complex understanding of identity and belonging in the world. Crenshaw’s theory has since been expanded to include the many overlapping layers of privilege and oppression that can exist within one individual. Intersectionality scholars encourage the study of overlapping marginalized identities as a method to understand the deeper issue of systemic and institutional oppression. Schuller (2022) states, “The experiences of marginalized people expose the true workings of power in all its forms. Identity forms a key piece of intersectionality, but it provides the lens, not the target (p. 7).

The ADDRESSING Model (Hays, 2022), uses an acronym which identifies ten different identity domains that can help to illuminate an individual’s position in society. The ten domains are age, developmental or acquired disability, religion, ethnicity, socioeconomic status (SES), sexual orientation, indigenous heritage, national origin, and gender. Each individual possesses their own set of identity domains, which interact and overlap in different ways resulting in individual variance of power and positionality within society. Bergkamp et al. (2020) proposed that the domain of ethnicity be expanded to also include racial identity, the domain of national origin be expanded to include citizenship, and that the domain of gender should include sex assigned at birth. Coupling the aforementioned definitions of social privilege with this understanding of intersectionality and positionality helps to illuminate how different identity domain combinations yield different levels of power in our world (Adams and Estrada-Villalta, 2019).

The terms agent and target can help us to further understand the complex phenomena of privilege and oppression (Adams et al., 1997). Agent refers to individuals who belong to the most socially privileged positions within a certain identity domain and target refers to individuals who belong to marginalized groups (Hays, 2022). For example, in a patriarchal society a cis-man is considered an agent and cis-women, trans people and non-binary people are termed targets. These terms intentionally evoke countervailing ideas of efficacy, control, passivity and victimization in order to highlight the stark power imbalance present in dynamics of privilege and oppression (Adams et al., 2018). This is further refined by Nieto’s (2010) distinction of rank versus status. While status refers to dynamic situations in which an individual might temporarily be in the minority (i.e., one of few white students in school), rank refers to the persistent aspects of identity that do not change across person, place, and situation. Further, rank is defined by historical intrenched systems and institutions that socially-confer or deny privilege based on identity domains (i.e., being Black or identified as a woman). However, it is critical to note that although individuals may experience the benefits of privilege and the detriments of oppression on a personal level, the forces of privilege and oppression are perpetuated on cultural and institutional levels (Nieto and Boyer, 2006). Thus, it is impossible for any one person to escape either force without substantially overhauling a myriad of societal systems (DiAngelo, 2016).

Furthermore, privilege must be seen as a corollary to oppression. Goodman (2015) refers to oppression and privilege as two sides of the same coin, and encourages individuals to shift their focus away from focusing solely on individuals who are disadvantaged, to placing an equal emphasis on studying those who are advantaged. She asserts that in order to truly understand the dynamics of social inequality that exists today in our world, we must understand the roles of *both* privilege and oppression and the way they mutually influence one another to produce and perpetuate prejudice and systems of oppression.

### Privilege in psychotherapy

Some recent psychological research is sensitive to the tension that differing levels of social privilege can cause in a therapeutic relationship and attempts to address privilege awareness within the therapeutic dyad (Suzuki et al., 2019). There is a burgeoning body of work explaining the potential pitfalls that may arise for therapists with agent ranks working with clients with target ranks (Fors, 2018; Hays, 2022). More specific research has corroborated these assertions by examining the negative impact of white therapists’ microaggressions on the therapeutic relationship with clients of Color and asserting white privilege as an essential concern of therapy (Davis et al., 2016; Hoffman, 2022). Similarly, it has been shown that when white therapists successfully initiate discussion about differences in privilege between themselves and their clients they strengthen their therapeutic relationships (Day-Vines et al., 2007; Zhang and Burkard, 2008; Edwards et al., 2017; LiVecchi and Obasaju, 2018; PettyJohn et al., 2020). Findings such as these lead to the creation of multiculturally sensitive ethical standards from the APA in an effort to protect clients from marginalized groups within the therapy room (Suzuki et al., 2019).

Recent research is beginning to consider the impact of social privilege on therapists with target ranks as well. A growing body of research examines the challenges faced by therapists dealing with direct discrimination from their clients (Haskins et al., 2015; Guiffrida et al., 2019; Branco and Bayne, 2020; Cottrell-Boyce, 2021). This focus is complemented by research examining therapists’ methods for dealing with client-expressed prejudices, which the therapist personally disagrees with, but by which they are not directly targeted (Spong, 2012; Drustrup, 2020).

Furthermore, such studies raise questions about therapists’ ethical duties to the broader community with regard to their decisions around interacting with prejudiced clients (Drustrup, 2020). The emphasis that these studies place on the experiences of clients is important, but there is a dearth of complementary research exploring therapists’ own experiences with social privilege. The present study aims to turn the mirror toward mental health providers, asking clinicians to consider their own understanding and experiences of social privilege. The aim of this study is to explore how social identity domains and areas of privilege and power may produce individual or relational stress in the therapeutic relationship.

Current research on social privilege within the context of psychotherapy reveals that we, as psychologists, have a strong tendency to study individuals who do *not* have privilege, rather than those who *do*. We posit that, perhaps by not researching the dynamics of holding social privilege within therapy, we are engaging in our own form of dysconsciousness (King, 1991). Turning a blind eye maintains a convenient status quo for those with privilege, since becoming aware of it can be an uncomfortable process (Bergkamp et al., 2022a,b). In fact, research has shown that social privilege awareness can elicit uncomfortable reactions: feelings of defensiveness and judgment, guilt or shame, and feelings of entitlement or a fear of loss, among others (Wise and Case, 2013). Despite this discomfort, researchers assert that we need to shift the narrative and focus on cultivating our own social privilege awareness as clinical psychologists (Black et al., 2007; Helms, 2017; Sue, 2017).

Engaging in the developmental process of our own social privilege awareness has many potential benefits. According to Black et al. (2007), counselors who possess privilege must become aware of this privilege in order to be considered truly competent. They assert that mental health providers must think critically about the consequences of oppression, and how they might consciously and subconsciously be enacting such oppression in their own work. Hays (2022) notes that in order to move toward cultural humility and competence, we must expand our understanding of multiculturalism to include notions of privilege awareness.

Sue and Sundberg (1996) observed that when therapists acknowledge differences between them and their client, it enhances counselor credibility, client satisfaction, depth of client disclosure, and clients willingness to return for follow up sessions. Zhang and Burkard (2008) noted that counselors who bring up differences are rated as more credible and have stronger working alliances with their clients than counselors who do not. Importantly, however, the authors noted that counselors of Color who bring up these differences with white clients do not see improvement ratings on credibility and working alliance. This underscores the fact that people working toward liberation frameworks are more often than not from marginalized groups themselves. Furthermore, this highlights the importance of making discussions of privilege awareness more mainstream among white counselors who hold immense power and privilege not only in the therapy room, but also in the world (Chavez et al., 2016). We believe that social privilege awareness should therefore be adapted into the core values of our work, and the core messaging of our training.

### Addressing gaps in current literature

Currently, there are no empirical studies that examine the perspectives of doctoral level clinical psychologists on social privilege awareness. The present study assumes that social privilege and oppression are corollary and divergent structural systems that are codependent and inseparable (Case and Cole, 2013). We integrate King’s (1991) notion of dysconsciousness to amplify how dangerous complicity can be, and to highlight how privilege often operates in invisible and unknown ways. We encourage readers to peel back the veil of oppression that dysconsciouness enshrouds us in, and expose ourselves to the rules of whiteness that dictate our behavior (Helms, 2017). We hope that by peeling back this veil, readers can be jolted into a state of critical awareness of their own power and privilege that may cause anxiety, confusion, and discomfort (Cottrell-Boyce, 2021). We also acknowledge the developmental nature of social privilege awareness (Bergkamp et al., 2022a,b), and anticipate that when we are willing to shed ourselves of privilege armor, we will likely feel uncomfortable, defensive, and protective of our values and integrity (Wise and Case, 2013). We acknowledge the work of scholars before us to bring this veil of oppression to the forefront of psychological research, and also acknowledge the lack of literature surrounding how understanding of and experiences with privilege impacts a psychologists’ personal experience in the field.

## Methodology

### Philosophical assumptions

We assert that contemporary systems of social privilege, which advantage certain groups over others, are a direct manifestation of historical colonization and the current coloniality mindset (Bhatia and Priya, 2018). Considering the aims of this project, it is important to scaffold the research with coherent philosophical assumptions that address issues of social privilege and avoid further coloniality in research. To this end, the researchers utilized inductive qualitative grounded theory with a social constructivist approach and an eye toward decolonization. The goal was to mindfully construct knowledge derived from the participant’s experience versus dictated from the more traditional colonial positivistic techniques that continue to center Western white patriarchy (Smith, 2012).

The colonial worldview valorizes historical European and contemporary American society as superior and universal by first commoditizing the idea of knowledge and then holding up traditional Western thought as the pinnacle of modernity and rationality. Thus, whatever “knowledge” those societies promulgate, be it the latest scientific research or capitalistic venture, is often perceived globally as the most correct and valuable knowledge available by virtue of the societies’ status at the top of the hierarchy. This type of artificially privileged knowledge also encapsulates more damaging and subtle colonial concepts like race and patriarchy (Quijano, 2007).

Coloniality is evident in the current reification of white, Western, male, heterosexual hegemony; and so contextualizes issues of race, sex, class, and gender-based discrimination within a historical context. Coloniality is a powerful influence on Western psychology’s definition of what is functional, normal, and healthy (Dirth and Adams, 2019). To reiterate, while colonialization is not a metaphor, and instead refers to historical atrocities, coloniality is the current manifestation of this legacy/history/context that permeates our daily lives (Tuck and Yang, 2012; Bhatia and Priya, 2018). According to Goodman et al. (2014), colonizing practices refer to those that “reproduce the existing conditions of oppression by failing to challenge the hegemonic views that marginalize groups of people, perpetuate deficit-based ideologies, and continue to disenfranchise the diverse clients and communities” (p. 148).

Antithetical to a colonizing perspective, a decolonial psychology emphasizes compassion over domination, generativity over stagnation, and distributive justice over the privileging of majority discourse, beliefs, and practices. Decolonial theory asserts that the human mind does not exist on its own, but rather, it exists within a rich and complex social context. As many scholars have noted, it is difficult, if not nearly impossible, to form one succinct definition of decolonial psychology. Instead, it is often depicted as a perspective shift that one adopts in an effort to resist dogma and instead welcome dissonance and disruption of epistemology and thought (Mignolo, 2011; Tate et al., 2013).

It is our belief that by adopting a decolonial, social justice and liberation-oriented stance, the APA will continue to adhere to its mission to “promote the advancement, communication, and application of psychological science and knowledge to benefit society and improve lives”(American Psychological Association (APA), 2022). By exploring the impact of social privilege on psychotherapy, we hope to shed light on the many visible and invisible impacts of colonial privilege on our profession.

### Research approach

#### Grounded theory

A grounded theory approach can be useful when limited research exists on a topic, as it allows the researcher to root a conceptual theory in the experience of those who actually confront the problem themselves (Charmaz, 2006). In grounded theory, the researcher is also incorporated explicitly as a subjective interactor in the production of knowledge, therefore situating the participants *and* the analysts as collaborators in knowledge production (Glaser, 1978; Creswell, 2018; Bergkamp, 2022). As a result, knowledge is digested in an emergent and rather never-ending process throughout the production of a theory. Consequently, grounded theory emerges as a transformative and developmental form of research which dedicates itself to the evolving act of resisting uncritical acceptance of one’s idea as a universal or explicitly known “truth” (Creswell, 2013).

Although some may claim it to be inductive in nature, Glaser (1978) asserts that grounded theory is both deductive and inductive; data collection, analysis and theory generation occur and reoccur repeatedly throughout the research process and constantly influence one another. When a code, category, or theme emerges, it is in turn compared with new and emerging data to determine the fit and relevance of the grounded theory. This in turn leads to increased theoretical sensitivity, or an enhanced ability to detect when concepts no longer “fit,” “work” or remain “relevant” to the data at hand.

## Method

### Study sample

Participants of this study were 9 psychologists over the age of 21 with doctoral level education in psychology. The demographic characteristics of each participant was assessed with a 9-item questionnaire based on the Hays’ (2022) ADDRESSING Model. All participants reported that they were natural born U.S. citizens. One participant reported being indigenous to a geographic or cultural region either by birth or by lineage. Five participants reported that they were white or European-American, two participants reported that they were biracial, and two participants reported being from a non-white, non-biracial racial identity domain. All participants considered themselves in middle to upper-middle class. Most (*n* = 7) participants reported falling between age 30 and 60. Most (*n* = 8) participants denied having either a physical or mental disability. Five of the participants reported subscribing to either Christianity or secularism within a primarily Christianity-influenced culture. One participant identified as a cis-gender male, with the remaining eight participants belonging to another gender identity domain. One participant identified as lesbian and eight participants identified as heterosexual.

### Recruitment

Before recruitment began, approval was granted for research with human participants by the affiliate university. The first author recruited participants using his own professional contacts. Then, further participant recruitment was completed using a snowball sampling method within participant’s professional networks. Participants were not given any compensation for their participation.

### Informed consent and assent

All participants were required to sign a written informed consent prior to being interviewed. Upon initial contact, each participant was provided with an electronic copy of an informed consent document which described the purpose of the project, the nature of the interviews, and any potential risk or harm they could be exposed to throughout their participation.

### Data collection

#### Procedures

Interviewers first obtained informed consent from participants, and then asked participants to fill out a short demographic questionnaire based on Hays’ (2022) ADDRESSING model, to collect uniform demographic data. Next, interviewers engaged volunteer participants with a set of 5 open-ended interview questions based on the overarching question of “what is your experience with social privilege within your psychotherapy practice?” These interviews were conducted either in-person, by telephone, or by video conference. Participants who participated in telephone or video conference interviews provided consent to being recorded before the interviews began. After each interview was completed, participants were given a list of crisis services in the case that the interview elicited distressing emotions. The researchers then stored the audio recordings on dual-password protected devices for later transcription. Researchers redacted personal information from each interview transcription, including participant names, names of spouses/children/family members, school affiliations, etc. After each transcription, all recordings were deleted immediately. Researchers then began qualitative analysis using the Dedoose computer software program.

### Data analysis

All data was analyzed using a grounded theory methodology, utilizing a constant comparative analysis (Glaser and Strauss, 1967). In line with grounded theory, the researcher is considered the main instrument of analysis (Bergkamp, 2010, p. 24). Therefore, we expected that the positionality and perspective of each researcher would have an impact on data collection, analysis and theory formation. To address this, we placed a heavy emphasis on researcher reflexivity throughout our data analysis process. The sequence of our analysis included open coding, focused coding, axial coding, and theoretical coding. Key concepts from the data were condensed to highlight main concepts, and then each of these concepts were compared and grouped into main categories. These main categories were then contextualized into a grounded theory understanding of how social privilege comes up in the therapy room.

## Results

**Figure 1:**
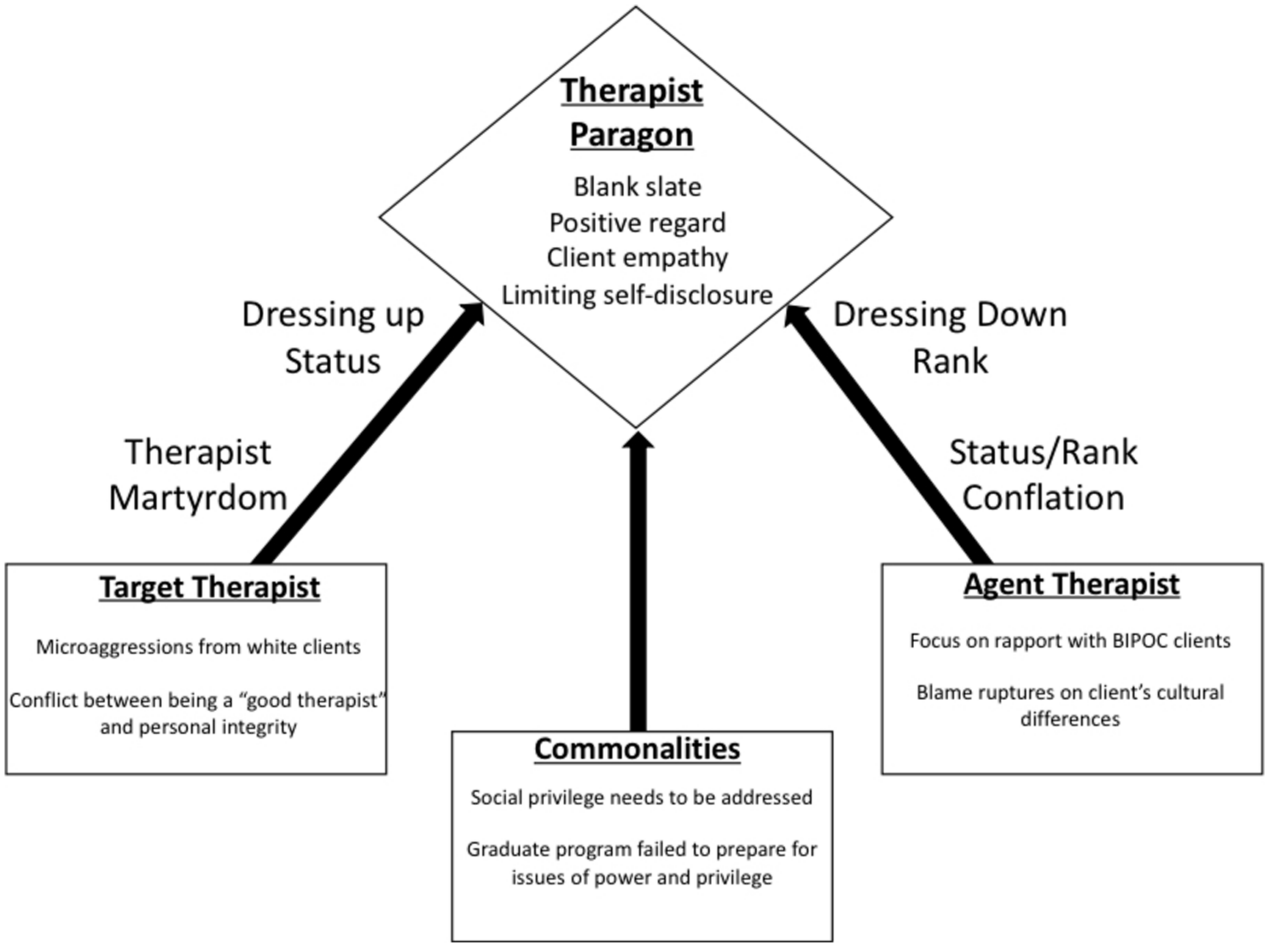
Pathways to the therapist paragon.

In this section, we will review the overall commonalities across the participants, as well as describe some important distinctions between participants. Namely, the analysis revealed a split between the white and BIPOC participants, with similarity among the two sub-groups and noticeable differences in comparison. Excerpts will be included to elucidate the themes. An abbreviated table of findings and next steps can be found in Figure 1.

### Commonalities

All participants conveyed that social privilege is a factor that impacts their psychotherapy. As a whole, they conveyed that being aware of the dynamics of social privilege was essential for therapy.

“I feel like the issue of privilege may be salient if the therapist hasn’t done enough work to know their own mind, to manage themselves, to - I mean certainly to be aware. I do see that as problematic.”

They also expressed a lack of related education during their graduate training, a lack of CEs offerings addressing this aspect in psychotherapy, or privilege being discussed in supervision or consultation. Most of the participants voiced some kind of confusion or difficulty when addressing this dynamic, especially as there seemed to be no clear guidelines to follow. In summary, all participants expressed concern regarding the right way to handle the issue of privilege in psychotherapy and a motivation to improve their professional services.

#### White psychologist participants

Most participants in this sub-group, when asked about their own social privilege, brought up examples of working with clients that were culturally different from them. None addressed how social privilege impacts therapy when the dyad match is both a white client and therapist. Furthermore, they identified common culturally-sensitive methods they employ to ensure initial rapport and therapeutic alliance.

“So, to me it is not so much about privilege, but it is about understanding the person and why they are the way that they are. And what is, you know, a phenomenological and empathic way to interpret their struggles and their behavior in a way that is non-judgmental and least critical and gracious.”

The unifying concept for the white participants was the emphasis on their own role power as the psychologist or expert, and the need to de-emphasize this power differential with the culturally-different client.

“You know, being a professional person with, you know, the title of doctor, there are certain social privileges that come with that. People have expectations. My own identity definitely has aspects of social privilege educationally.”

“So, first of all, I have the privilege of being a clinician. And I’ve been benefitted or given certain access to education and financial support to be able to do what I do. And so, when I’m working with clients or patients most of them don’t have the same opportunities that I’ve had.”

“The issue [of privilege] psychotherapy practice that transcends social and cultural identity is that the reality of the power differential of the provider and the patient and the client is extreme. And I think that it’s underestimated a lot. People don’t necessarily take that as seriously as they should.”

These included attempts to equalize power within the relationship and efforts to emulate the clients assumed cultural norms for the sake of rapport. Examples include attempting to emulate the assumed cultural practices of the client, and adopting a collaborative and strengths-based approach to counteract their client’s societal marginalization. Some mentioned dressing more plainly, using first names, and avoiding talking about themselves when having sessions with clients that held marginalized identities. They also attempted to speak in simple English, limiting professional jargon, instead working to adopt cultural phrases that might put the client at ease.

“One of the things that I think we all struggle with a little bit is what’s the best way to present ourselves in a clinical setting? I have the notion that I wanted to make myself, I didn’t want to accept the authority of the position in some way and so if I dressed more casually, be more relaxed, use more slang or colloquial language, this would make me less threatening, or more like a peer and that the way to build a therapeutic alliance was in some ways to be a peer and think about that and have a patient feel more comfortable. I realized that that didn’t fly. I was still the therapist.”

When the issue of therapeutic rupture and repair with culturally-different clients arose in the interviews, white participants seemed to attribute the difficulty to the lack of client readiness or capacity for change. There was no consideration of the cultural values of the client, or the possibility that western psychotherapy could be a foreign cultural concept for the client. Also lacking was the potential for cultural assumptions or tendencies to pathologize cultural difference due to the psychologist’s implicit bias. If there was some consideration of culture, there was a lack of acknowledgement that the therapist’s whiteness may contribute to this dynamic.

“Again, I’m not sure that those identity factors trump an actual psychotherapy relationship where the therapist has a lot of power as the expert, as the person who’s helping, as the person who’s getting paid. I’m not convinced that the social dynamic is any more, but I also have never been any - I’ve never been a man, I’ve never been a Person of Color (laughs), so I don’t know if I would feel differently about that if I were a therapist who was, you know, different.”

Expanding on their challenges when working with clients who are culturally-different, especially BIPOC clients, the white clinicians also brought up the limited multicultural education in their graduate programs and the lack of clear practice guidelines. They felt uncomfortable addressing ruptures and most doubled down on ideal therapeutic values of forgoing their own feelings and keeping the attention on the client to preserve rapport and alliance.

“I think it’s problematic, and in this case it was tricky because I knew there was stuff going on with racial identity for this adolescent girl who was seeking out her white parent, like curious about her white parent. But this African American couple said “oh no, it’s just not an issue in our family.” I felt kind of in a bind about being able to say “oh yes it is!” (Laughs) I didn’t even really feel like I could say “really?” which would have probably been the best intervention right then, like “huh? I’m confused!” you know?”

#### BIPOC psychologist participants

When this group was asked about the impact of social privilege on psychotherapy, the participants focused on challenges that arise when working with white clients. While they mentioned the essential need for cultural humility and effective practice, including clinician self-awareness, most of their narrative involved personal and professional risks and costs when working with white clients. The common themes for this sub-group involved microaggressions from the white client toward themselves as the psychologist. Confusion regarding how to therapeutically respond during therapy. The struggle to establish credibility with their clients and colleagues. And compensation strategies used regarding the microaggressions and threats to professional credibility.

“I remember one of my first clients when I was a trainee. Oh my god, it was so awful. It was a white couple, I think they were. And they just really didn’t like each other, so they were a difficult couple. And they didn’t want to work with me. And I remember feeling very upset. They didn’t want to work with me because they thought I was the…there was a sliding scale, I was the trainee, and they came to the clinic because I presume it was a clinic, and there was this racialized way they coded it. They coded it as, like, they didn’t want the discount therapist. I think that’s the word they used, the discount therapist. And I knew it was because of race. We were all discount therapists, you know.”

The most striking similarity among this sub-group was the reports of microaggressions from white clients and colleagues. These microaggressions came in many forms but usually involved questions about the veracity of the BIPOC psychologist’s education, training, experience, and expertise. Participants also expressed concern and confusion for how to address microaggressions during therapy. They stated that they had not been trained for these kinds of situations, especially when the dyad is a white client and a BIPOC therapist. They were nervous about appearing too focused on their own feelings at the sake of the client’s therapeutic needs. Most tried to ignore these events and some were concerned that bringing up the possibility of microaggressions with their clients could result in the client not showing up again.

“I have another client who sticks out to me, they were somebody who I thought did good work in the community, but she was from a much higher SES bracket than me, even today, I think for generations, she had generational wealth. Issues came up in her life around the business that she was in and I said something, I don’t remember what, but I recognized that she had more social privilege than me, and then she fired me, or eventually she just didn’t come back I guess.”

These microaggressions would occur at all stages of the process, from clients hiring of the therapist, to the initial sessions, all the way through to termination. This caused the participants to question their own status and role, along with their ability to live up to the expectations of a good therapist. A few participants questioned if the microaggressions were due to the fact that they may not look, sound, or act like the quintessential therapist that the client expected. Another main construct that permeated this sub-group data was the constant efforts to seek credibility from clients, colleagues, and from themselves. Much of this process was triggered by microaggressions from clients and colleagues, but also due to imposter syndrome. This was accompanied by worries about how they are being perceived and what kind of judgmental criteria was being utilized.

“I was very new and self-conscious. I already felt like I wasn’t good enough. I was already like “I don’t know how to do this therapy stuff,” and these people didn’t want me because I’m not good at therapy. Of course I’m not good at therapy. So they played into the fear that I understandably had. I was also quite young. So they were older than me, they were white. They weren’t old, but they were maybe in their late twenties, thirties, I started graduate school pretty young. I was in my early twenties. So they were older than me, but not old. They were white, and they felt insulted that they got the “discount therapist,” I remember that. So I definitely felt targeted racially. That was really horrible.”

BIPOC participants reported different ways of dealing with microaggressions and imposter syndrome. Some would experiment with bringing it up during therapy, which they felt would violate norms of neutrality or personal self-disclosure. Some were impacted to the degree in which they decided to only see BIPOC clients or stopped psychotherapy altogether, opting for evaluations that did not demand the same level of rapport and alliance, or transition to academia or consulting. For those that continue engaging in psychotherapy, they reported constantly engaging in continuing education to substantiate their expertise to both clients and colleagues. They purposefully hang all of their diplomas and certificates on the wall, make sure that their offices are well furnished and they dress as professional as possible, and highlight their most recent CE session. All in a way to reinforce their status as the expert to be trusted.

## The grounded theory: pathways to the therapist paragon

As a reminder, the core analytical question during the grounded theory analysis is identifying the dilemma experienced by the participants and the process in which they deploy to address and resolve this dilemma. In this study, the participants were asked about the construct of social privilege, how it impacts their work as psychotherapists, and what they do to address this process.

### The core concept–therapist paragon

The core concept that appeared to pervade much of the data was the Therapist Paragon. The word paragon is defined as “a person or thing regarded as a perfect example of a particular quality” (Oxford English Dictionary, 2022). The concept of the Therapist Paragon can be defined as the internalized referential image of a perfect therapist, one that practicing therapists use to evaluate themselves and other therapists. It consists of the underlying values and responses that a perfect therapist is supposed to embody. Usually including a clinician’s overall motivation to help others through deep connection and sophisticated guidance, a full focus on the client’s wellbeing and healing, and an ability to set themselves aside and remain emotionally grounded for the sake of the therapeutic alliance. This last value also encompasses the issue of self-disclosure and the need to sustain a fairly neutral stance during the therapeutic process.

The Therapist Paragon is utilized to establish when the individual therapist believes they have done a good job, or not, during and after the session. When they believe their performance was not satisfactory, it usually means they did not reach the paragon standard. The therapist paragon consists of idealized predecessors of the field, the ethical aspirations, internalized mentors and past supervisors, and the underlying values of prominent theoretical orientations. While the origins of a professional’s Therapist Paragon can begin to take root in early personal therapeutic experiences, the formation and foundation occurs during educational training. The modification can occur with subsequent continuing education and consultation/supervision, but usually this does not significantly impact the core paragon beliefs and values.

Psychotherapy education for professional clinicians include the teaching and practice of person-centered therapy, evidence-based common factors of effective psychology, and the remnants of psychoanalysis. The underlying values innate in these approaches include the therapist as a blank-canvas for client projections, the intentional use of self-disclosure, the emphasis that every effort is made for the client’s well-being or treatment, and a general bracketing of the clinicians personal needs and reactions. Rogers (1951), a pillar of the field, encouraged clinicians to exercise unconditional positive regard, clinician authenticity, and empathy in a non-directive format that prioritized a self-actualization framework. Taking this approach further, the recent common factors literature makes the empirical assertion that therapeutic alliance, therapist empathy, position regard, and genuineness are essential for effective therapy (Norcross and Lambert, 2018).

The remnants of psychoanalysis also find their way into contemporary curriculum. Psychoanalytic concepts of disclosure, projectives, analysis free of bias, interpretation, and passivity continue to be taught and practiced today. This also includes the psychoanalytic concept of neutrality, which encourages the therapist to resist the natural urge to reciprocate affect and analyze transference as opposed to responding to it (Malcolm, 1981). Malcolm (1981) described psychoanalysis as voyeurism, asserting that one watches what is happening, but does not jump into the fray.

These, and other common approaches taught in professional training, constitute the underlying beliefs and values of the Therapist Paragon. This common curriculum is usually applied equally to all psychotherapy students, without much consideration for their social location, socially-conferred privileged, or oppressed identities. The pioneers of the field consist predominantly of white theorists and practitioners, with most also being male. A more critical pedagogical approach to training usually results in the ingratiation of white, male normative hegemony seeping into the field, resulting in the Therapist Paragon. Again, an internalized metric of good or bad performance and being - one that white, and male, therapists can find more personal congruence within.

#### The blank-slate value

The blank-slate value innate in the Therapist Paragon makes seamless therapeutic alliance an epitome of practice, ostensibly to avoid harm to the client. This core concept suggests this epitome is unattainable and may cause harm to the therapist, and leads us to question whether it is always entirely beneficial to the client. When a blank-slate therapeutic alliance is seen as the standard, therapists learn there are limits to their authenticity. They must be authentic enough to establish rapport, but not so authentic that conflict arises in the therapeutic alliance. This means therapists may need to deny their true selves when they are experiencing harm in the relationship. The blank-slate therapeutic alliance says harm to the therapist does not matter; only harm to the client matters. This virtually ensures the therapy room will be a place where therapists with target ranks will need to code-switch, denying their own position in a system of unequal privilege and power in order to play their role (Sithole, 2022). Therapists with agent ranks, on the other hand, benefit from the blank-slate therapeutic alliance, since it provides a built-in reason—avoiding client discomfort or harm—to maintain dysconsciousness and avoid addressing the harm all people experience in a system that maintains the privilege and power of some at the expense of others.

Because the status quo itself leads to harm, there is a question whether the blank-slate therapeutic alliance is entirely beneficial to clients. Clients may benefit from authentic therapeutic alliances that can weather rupture and repair, and clients may find growth in discomfort and appropriate challenge to their beliefs and behaviors. An authentic, conscious therapeutic alliance may prove more engaging and beneficial to clients than one in which the therapist performs for the client as a dysconscious or code-switching paragon.

#### Pathways to the therapist paragon

The most notable dynamic within the data was the differences between the white and BIPOC psychologists that participated in the study. Specifically, they both alluded to the Therapist Paragon, in that they discussed underlying values they strive for to make their services effective. The Therapist Paragon beliefs and values were strikingly similar across both sub-groups. Yet, the pathway of each group toward the therapist paragon was distinctly different. Both in terms of strategies used to obtain perfection and client approval, as well the impacts to their professional and personal selves.

### White and BIPOC pathways

Commonalities across the sample was a notion that social privilege does impact psychotherapy and it is important to address in some way. The core concept that united all the participants was an underlying pursuit and adherence to their internalized image of the perfect therapist, embodying core values in psychotherapy. The therapist’s core values most applicable to their dilemma was to prioritize the clients needs first by setting themselves (feelings, thoughts, beliefs) aside for the sake of the client, the use of self-disclosure, remaining non-judgmental, assuming the best intentions, and maintaining empathy. The core category of the Therapist Paragon was mentioned or alluded to by the participants in view of the fact that they strived to provide adequate care, assess their own performance, their approach to consultation with colleagues, and navigate the therapeutic relationship.

The core differences between the sample seemed to generally occur down the line of white and BIPOC psychologists, with each sub-group expressing common themes. The most striking sub-group differences regarding social privilege involved the therapeutic dyad focus, methods used to establish and maintain rapport and alliance, and their personal experience in these interactions. Thus, the pathway toward the Therapist Paragon was paved and reassuring for the white sub-group or the source of harm and self-doubt for the BIPOC sub-group.

### White psychologist sub-group

#### Status/rank conflation

One mechanism this sub-group utilized to pursue the Therapist Paragon was termed “status/rank conflation.” While the white participants focused heavily on their status power as a “doctor” or “expert,” most did not acknowledge their own social location or privileged identities, possibly due to Therapist Paragon values. This can be explained by the conceptual distinction of status versus rank (Nieto, 2010). Status is a state-like dynamic role that may hold temporary privileges within certain contexts, such as being a psychologist, but is not permanent across settings. Rank is a static trait-like aspect, such as being a BIPOC individual, that is consistent across time, person, and situation (Hays, 2022). The convenient confusion when asked about privilege was to focus on their educational and professional status versus their whiteness.

#### Dressing down rank

The other Therapist Paragon mechanism for this group was “dressing down rank.” The white group discussed cases with a culturally-different other, presumably working with BIPOC clients. They focused on how they could modify their appearance and behavior to make rapport/alliance easier, attempting to match the client’s culture. They tried to embody a friend or confidant role by ignoring the power dynamic and emphasizing equality. They relied on their multicultural education, working to suspend assumptions and maintain a self-less approach. When difficulties arose, they attributed these to the cultural differences innate in the client. Other participants asserted that the lack of their cultural knowledge was due to lacking multicultural education in their graduate programs, versus dynamics of their own social privilege awareness. In the case of the data, the white participants discussed the interpersonal dynamics of status privilege, but did not address their socially-conferred privilege as white individuals.

Ultimately, when involved in this white therapist/culturally-different client dyad, this sub-group replicated the Therapist Paragon they were ingrained with throughout their professional career, usually that of a white male. The values of an ultra-focus on the client, minimizing self-disclosure, and modifying themselves for the sake of the client may also allow the white therapist to avoid the truth of social privilege power differences within the therapeutic relationship. It fosters a sense of confidence in their intention without a full awareness of their social location and the inevitable impacts.

The Therapist Paragon values can serve as a method of transcendence from the worldly constructs of race, relying fully on unconditional positive regard and non-judgmental empathy. For the white psychologists, the pathway to the Therapist Paragon was paved by the congruence of whiteness. The congruence of whiteness between themselves and the paragon image allows for an assurance they are doing the right thing, and when things do not work out, it is not a reflection of privilege differentials, but rather the cultural aspects of the client.

### The BIPOC psychologist sub-group

In direct contrast, the BIPOC sub-group discussed microaggressions from clients and colleagues, the internal struggles with imposter syndrome, and the common compensation strategy of bolstering their status power as the doctor and expert.

### Therapist martyrdom

In the face of microaggressions from white clients, the paragon values of setting themselves aside and unconditional acceptance prevented them from speaking up in the spirit of mutual respect. It, instead, resulted in them being complicit in their own harm for the sake of the therapeutic relationship or the real fear of losing clients and the risk of a negative reputation in the professional community. The paragon values reinforced the societal pressure for BIPOC professionals to sacrifice their own needs and dignity in pursuit of the ingrained image of white perfection.

### Dressing up status

Their compensation included attempting to work harder compared to their white counterparts, attempting to appear competent and professional, and constantly accrue knowledge through certifications and continuing education sessions. Due to the persistent distress caused by these dynamics, some of this sub-group made the conscious decision to stop working with white clients or psychotherapy altogether.

The primary method in which this sub-group strove toward the Therapist Paragon was by emphasizing their education, training experience, and expertise. This was done by attempting to accumulate more knowledge than white colleagues and appearing more competent in the way they spoke, dressed, and displayed their diplomas and certificates. This “dressing up status,” as termed during analysis, was an effort to counteract the microaggressions and imposter syndrome they experienced throughout their career.

For these participants, the pathway to the Therapist Paragon was paradoxical, in that it appeared to cause pervasive feelings of self-doubt and personal harm. The paragon values allowed the microaggressions and imposter syndrome to thrive and isolate this sub-group. While it appeared to guide the white therapists through complex issues of power differences for the sake of their own comfort, the ingrained image of white, male therapeutic perfection resulted in added burden for the BIPOC sub-group.

## Discussion

The implications of this grounded theory involves all aspects of the human service field. For the sake of brevity, this section will focus on clinical and counseling psychology with an initial attempt to examine graduate education and continuing education, as well as supervision/consultation. We also question if these paragon values are codified in our ethics codes. Specifically, we call on the field to consider the origins of the Therapist Paragon and how this image of therapeutic perfection impacts clinician’s careers across racial lines.

### Graduate training

The proposed definition of the Therapist Paragon is the constellation of implicit and explicit principles and values regarding what makes good therapy and how the perfect therapist is supposed to look, sound, and act. Sources of this internalized image of perfection consist of theories, research, guidelines, and common practice. These are introduced and ingrained during graduate education in the form of textbooks, articles, lectures, role plays, clinical training, supervision, and evaluation. Thus, through graduate school, the clinician is gradually formed into the field’s image of the competent entry-level practitioner.

Considering the history of our field, many of the prominent forbearers are white and male. These theorists and practitioners are closely studied and honored through reading, discussing, and emulating their techniques. While the field educates its students in theory and practice, it is difficult to separate the ideas and techniques from the originating pioneer. Thus, we naturally celebrate both the pioneer and the knowledge. While there have been many brilliant BIPOC scholars and clinicians throughout the history of psychology, they usually are underrepresented in the curriculum. Furthermore, while most programs include a class addressing individual and cultural differences, many do not include the aspect of socially-conferred privilege and how to account for this in psychotherapy.

The implications of this study point to the possible negative impact upon both BIPOC therapists and clients due, in part, to the Therapist Paragon. To address this, further research and programmatic changes are recommended. Specifically, decolonizing the curriculum has been a recent trend in some pockets of psychology (McCubbin et al., 2023). This usually involves adopting a critical lens to examine how current education is perpetuating a white supremist, patriarchal view of the field based on historical practices (Ratts et al., 2016). Efforts to reframe these aspects of the syllabi include re-centering international, indigenous, and BIPOC scholars into the curriculum, acknowledging the field’s historical contribution to oppression of marginalized groups, and daring to consider new ways of research, assessment, and practice (Ponce et al., 2023). Additional recommendations include increasing diversity within faculty and students, and considering the impact of cultural competence and humility training on both white and BIPOC students.

### Continuing education

Continuing education is a common mechanism to keep practitioners trained in the most contemporary research and practice of the time. It is an embodiment of the professional value of lifelong learning in the field of psychology. In light of these results, it is important to consider that the Therapist Paragon can be deeply ingrained in the learner, especially dependent on what era they attended graduate school. Much of multicultural education has changed drastically over the decades, with the focus on social privilege and social responsiveness occurring in recent years (Bergkamp, 2022). Thus, it is important to consider the learner’s era of training and focus on updating their knowledge from that point to develop a congruent historical sequence. This will also assist in understanding possible philosophical conflicts in supervision and consultation.

In addition, current continuing education offerings need to match the contemporary curriculum of graduate programs. As aforementioned, both need to expand the training from cultural differences within the therapeutic dyad to historically entrenched systems of social privilege allotment that impact psychological functioning (Wilcox, 2022; Bergkamp et al., 2022a,b).

### Ethics

All of our professional associations within the field of psychology include an ethics code that guide and mandate education, research, and practice. The origins of these ethics consist of commonly held beliefs and values of psychology that increase benefit and mitigate harm. In light of the Therapist Paragon theory, it is hypothesized that our ethics codes are a potent source of therapeutic perfections that may be embedded within a history of hegemonic colonial values. Further research and consideration is encouraged to assess the codification of white, patriarchal norms embedded within our aspirational principles and requirements.

## Data availability statement

The raw data supporting the conclusions of this article will be made available by the authors, without undue reservation.

## Ethics statement

The studies involving human participants were reviewed and approved by Antioch University Seattle. The patients/participants provided their written informed consent to participate in this study.

## Author contributions

All authors listed have made a substantial, direct, and intellectual contribution to the work and approved it for publication.

## Conflict of interest

The authors declare that the research was conducted in the absence of any commercial or financial relationships that could be construed as a potential conflict of interest.

## Publisher’s note

All claims expressed in this article are solely those of the authors and do not necessarily represent those of their affiliated organizations, or those of the publisher, the editors and the reviewers. Any product that may be evaluated in this article, or claim that may be made by its manufacturer, is not guaranteed or endorsed by the publisher.
